# Ambipolar Transistors Based on Vertical Heterojunctions for Multivalued Logic Gates

**DOI:** 10.1002/smsc.70389

**Published:** 2026-11-04

**Authors:** Ke Zhou, Shengnan Liu, Yucai Yuan, Jiawei Hong, Zhixiang Ren, Yaxiao Lian, Xiaotao Zhang, Wentao Xiong, Chen Zou, Baodan Zhao, Wenping Hu, Dawei Di

**Affiliations:** ^1^ State Key Laboratory of Extreme Photonics and Instrumentation College of Optical Science and Engineering International Research Center for Advanced Photonics Zhejiang University Hangzhou P. R. China; ^2^ Tianjin Key Laboratory of Molecular Optoelectronic Sciences Department of Chemistry School of Science Tianjin University and Collaborative Innovation Center of Chemical Science and Engineering Tianjin P. R. China; ^3^ College of Chemistry and Chemical Engineering Xiamen University Xiamen P. R. China; ^4^ ZJU‐UIUC Institute Zhejiang University Haining P. R. China

**Keywords:** ambipolar transistor, multivalued logic, ternary inverter, vertical heterojunction

## Abstract

Multivalued logic (MVL), which encodes information beyond binary states, offers a transformative approach to enhancing computational efficiency in the post‐Moore era. Conventional MVL implementations typically rely on lateral heterostructures that exhibit negative differential transconductance (NDT) or negative differential resistance (NDR) behavior. However, such designs are often hindered by fabrication complexity and sensitivity to voltage thresholds. Here, we present a simple and effective strategy for MVL devices: a vertical heterojunction architecture with a self‐assembled monolayer (SAM)‐modified interface. The SAM not only passivates surface defects but also spatially confines hole carriers. This yields a distinctive V‐shaped transfer characteristic with suppressed hole conduction, a feature critical for achieving stable intermediate logic states without the need for intricate multithreshold engineering. We further demonstrate a ternary inverter with three distinct logic states, whose intermediate state range is dynamically tunable via supply voltage in the subthreshold regime. Our work provides a promising pathway toward energy‐efficient MVL systems for applications in artificial intelligence and edge computing.

## Graphical abstract

We demonstrate a vertical heterojunction ambipolar transistor using a self‐assembled monolayer (SAM)‐modified interface that suppresses hole conduction and creates a distinct V‐shaped transfer characteristic. This design enables a ternary inverter with three stable logic states, tunable in the subthreshold regime, offering a simplified and efficient pathway toward energy‐efficient multivalued logic for AI and edge computing.

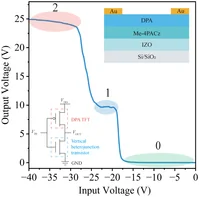

## 1 Introduction

Multivalued logic (MVL), which encodes information using three or more distinct states (e.g., ternary, quaternary), has re‐emerged as a transformative paradigm for advancing semiconductor technology in the post‐Moore era [1, 2, 3, 4]. Its significance stems from the potential to overcome critical limitations of CMOS technology, particularly the power‐density wall and interconnect bottlenecks. By encoding multiple bits of information per signal input, MVL substantially reduces the physical complexity and electrical loading of interconnects, directly addressing RC delay constraints and minimizing dynamic power dissipation. These advantages prove especially crucial for emerging computational‐intensive applications such as artificial intelligence (AI), edge computing, and the Internet of Things (IoT), where stringent requirements for energy efficiency and hardware compactness dominate system design priorities [5, 6].

Conventional MVL implementations often rely on lateral heterojunctions [7, 8, 9]. Recent advances leverage emerging materials such as organic semiconductors, van der Waals (vdW) heterostructures, and oxides to engineer such heterojunctions via phenomena like negative differential transconductance (NDT) [10, 11, 12], negative differential resistance (NDR) [13, 14], or antiambipolar behavior [15, 16], enabling nonmonotonic current–voltage characteristics essential for MVL operation. Featuring transfer curves with diverse shapes (N, W, Λ, etc.), these devices successfully demonstrate core functionalities including ternary inverters [17, 18, 19]. However, lateral structures present inherent fabrication challenges, such as precise alignment of dissimilar materials, complex patterning steps, and compromised interfacial control. Furthermore, MVL operation using ambipolar transistors has been achieved through channel doping or special treatment [20, 21], yielding W‐shaped transfer curves with multiple threshold voltages (multi‐*V*_TH_) to define logic states. The intermediate state exhibits sensitivity to process variations, particularly multi‐*V*_TH_ fluctuations. Nevertheless, realizing MVL functionality with vertical heterojunciton ambipolar transistors remains an unresolved challenge.

To address this limitation, we introduce a novel MVL device architecture that significantly simplifies implementation. Our approach centers on a vertical heterojunction structure, distinct from conventional lateral configurations, offering inherent advantages in structural simplicity, enhanced electrostatic control, and streamlined fabrication. Crucially, this architecture enables ternary logic operation with voltage‐tunable transition between three‐state and two‐state behavior, thereby eliminating the need for complex multithreshold engineering. By synergizing the operational efficiency of ambipolar transport with the manufacturability of vertical integration, we realize a ternary inverter exhibiting three well‐defined states. This approach establishes a promising pathway toward practical MVL unit devices, directly overcoming critical barriers associated with multithreshold designs and complex lateral heterostructures.

## 2 Results and Discussion

As illustrated in Figure 1a, we present a simple and effective strategy for realizing MVL devices through a vertical P–N heterojunction architecture featuring self‐assembled monolayer (SAM)‐modified interface. Indium‐zinc oxide (IZO) was selected as the n‐type semiconductor owing to its superior film uniformity, stable amorphous structure, and compatibility with solution‐processed materials [22, 23]. For the p‐type active layer, 2,6‐diphenylanthracene (DPA) was employed due to its high hole mobility and superior environmental stability (Figure 1b) [24, 25]. To optimize interfacial properties, (4‐(3,6‐dimethyl‐9*H*‐carbazol‐9‐yl)butyl)phosphonic acid (Me‐4PACz) was introduced as the SAM, serving dual functions: passivating surface defects on the IZO substrate and facilitating hole carrier accumulation and transport [26, 27]. The typical transfer curves and output curves of the MVL devices are shown in Figures 1c,d and S1 at various drain–source voltages (*V*_DS_). The devices exhibited n‐type‐dominated ambipolar behavior, with a minimum drain current (*I*_D_) occurring at a gate voltage (*V*_G_) of −10 V. Initially, the *I*_D_ saturates within the *V*_G_ range of −100 to −40 V at *V*_DS_ = 10 V, attributed to hole accumulation in the DPA layer. As *V*_G_ is swept toward positive values, *I*_D_ undergoes a linear reduction followed by a pronounced recovery at higher *V*_G_ regimes. This behavior indicates concurrent hole carrier depletion and electron accumulation—a hallmark of ambipolar charge‐transport dynamics. Beyond this transition threshold, electron‐dominated conduction prevails in the channel, establishing unipolar n‐type operation at elevated gate biases. Increasing *V*_DS_ raised the minimum *I*_D_ by over an order of magnitude (from 8.6 × 10^−8^ to 3.6 × 10^−6^ A) and induced rapid *I*_D_ saturation, indicating pronounced ambipolar transport. As shown in Figure 1d, the output curve also reveals that *I*_D_ remains independent of *V*_G_ within the range of −100 to −60 V.

**FIGURE 1:**
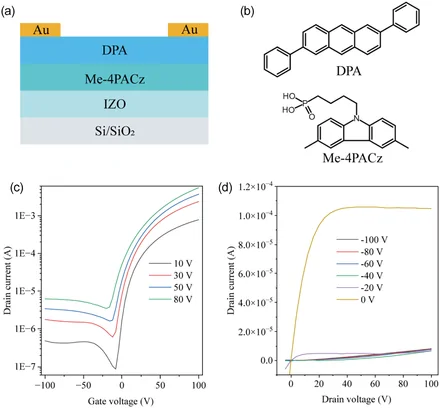
(a) Schematic of the vertical heterojunction ambipolar transistor. (b) Chemical structures of the p‐type organic semiconductor DPA and the self‐assembled molecule Me‐4PACz. (c) Typical n‐type transfer curves and (d) output characteristics at varying drain–source voltages.

The n‐type IZO layers were deposited onto the SiO_2_ substrate via spin‐coating. Atomic force microscopy (AFM) was used to examine the surface morphology of the resultant IZO films, which were amorphous and uniform with a root‐mean‐square (RMS) surface roughness of ~0.5 nm (Figure S2). Despite their smooth morphology, the amorphous IZO surfaces contained hydroxyl groups and exhibited high surface energy, which detrimentally impacted hole transport in the upper p‐type channel. To address this, the IZO films were functionalized with a Me‐4PACz SAM. This treatment passivated surface hydroxyl groups through phosphonate bonding and reduced surface energy [28], thereby facilitating the subsequent deposition of DPA thin films. Subsequent deposition of the DPA layer on both SAM‐modified and unmodified IZO substrates revealed a Volmer–Weber (island) growth mode. The SAM‐modified interface exhibited low surface energy, which typically facilitates DPA thin film formation, but the resultant DPA films showed reduced grain sizes and enhanced morphological homogeneity. This is supported by a decrease in RMS roughness from 27.9 to 18.3 nm (Figure S3). X‐ray diffraction (XRD) analysis further indicated reduced crystallinity in DPA films deposited on SAM‐functionalized surfaces, as demonstrated by diminished diffraction peak intensity relative to films on bare IZO substrates (Figure S4).

To investigate the origin of the *V*_G_‐independent *I*_D_ phenomenon in this architecture, we fabricated and characterized SAM‐free devices using field‐effect transistor (FET) measurements (Figure S5). At least 10 devices were tested for each condition. As shown in Figure 2a, compared with SAM‐modified devices, SAM‐free devices exhibited exclusively unipolar n‐type characteristics. This occurs despite ohmic contacts between the DPA layer and gold electrodes in both architectures, which theoretically facilitate hole injection. The unexpected unipolar behavior implies that the *V*_G_‐independent *I*_D_ phenomenon likely originates from the IZO rather than the DPA layer, even under hole accumulation‐dominated channel conditions. Typically, larger grains and higher crystallinity are beneficial for charge transport in organic semiconductors, as they reduce grain boundary scattering. One would therefore expect SAM‐modified devices to exhibit degraded hole transport. However, our electrical measurements reveal that SAM‐modified devices actually show markedly enhanced hole transport characteristics. These results indicate that although SAM modification leads to reduced grain size and lower crystallinity in the DPA film, it is nonetheless essential for enabling efficient hole carrier accumulation and transport in the vertical heterojunction system. To further investigate the role of the SAM, we fabricated devices with varying SAM concentrations. As shown in Figure S6, increasing the SAM concentration led to a random distribution of nanoscale domains across the SAM‐functionalized IZO surface. This morphology can be attributed to the self‐organization of the intrinsically amphiphilic SAM molecules into micellar structures during deposition. Corresponding electrical characterization (Figure 2b,c) revealed that all devices exhibited n‐type‐dominated ambipolar transport behavior. A higher SAM concentration resulted in a significant decrease in the *I*_on_/*I*_off_ ratio, along with an increase in both hole and electron mobility. These results confirm that the concentration of 0.5 mg/mL represents the systematically optimized condition for the SAM, which yields the most pronounced ambipolar transport characteristics. Furthermore, we fabricated DPA thin‐film transistors using a SAM‐modified SiO_2_ gate dielectric. The transfer curves demonstrated good linearity over a wide gate‐voltage range (Figure S7). The average hole mobility, calculated from the slope in the saturation regime, was 0.27 cm^2^ V^−1^ s^−1^. These results suggested that the suppressed hole conduction in the vertical heterojunction transistors was not primarily attributable to the DPA or SAM layers.

**FIGURE 2:**
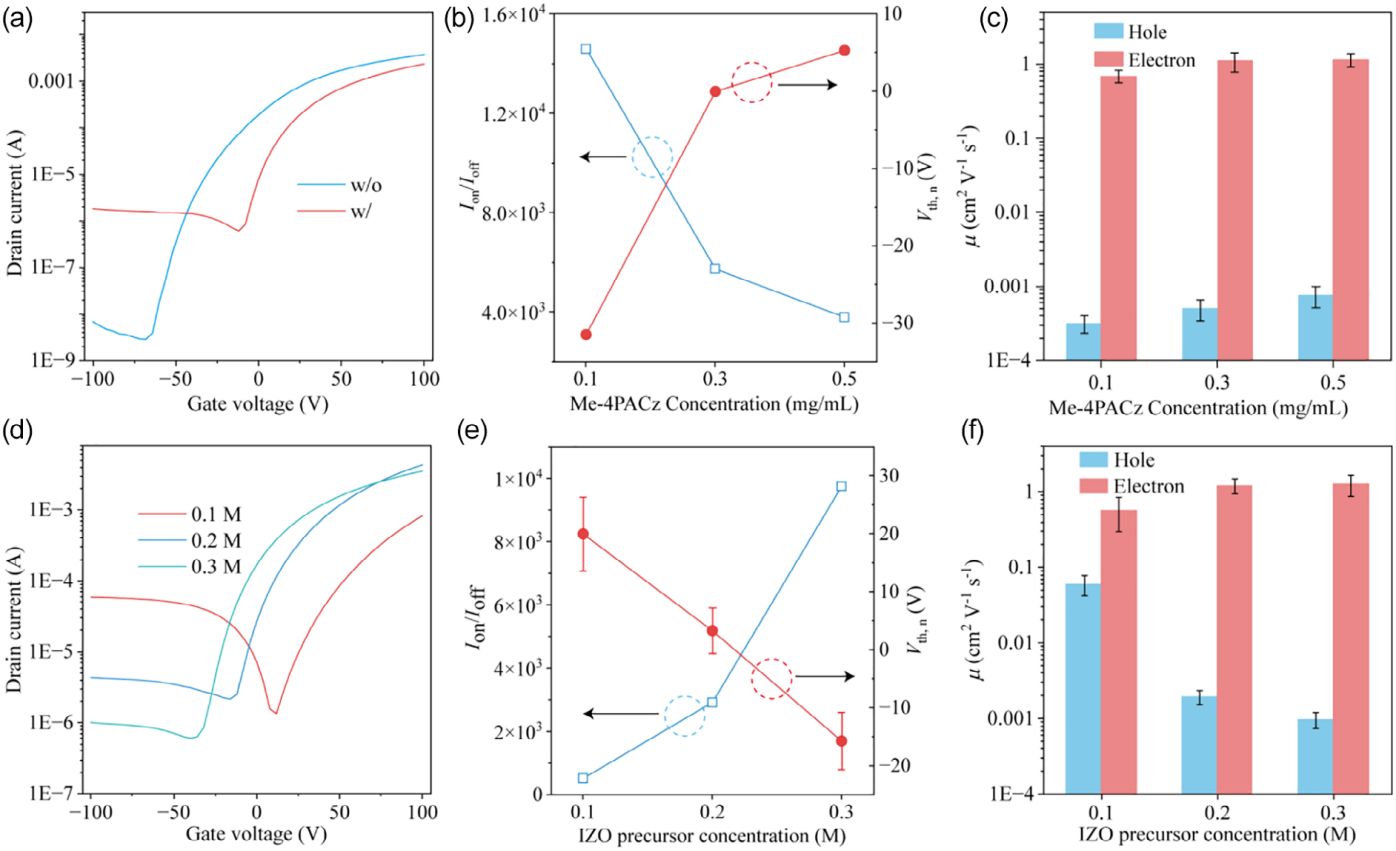
(a) Transfer curves of vertical heterojunction transistors with and without SAMs modification. (b) *I*_on_/*I*_off_ and threshold voltage as functions of Me‐4PACz concentrations. (c) Hole and electron mobilities of vertical heterojunction transistors at various Me‐4PACz concentrations. (d) Transfer curves of vertical heterojunction transistors at various IZO precursor concentrations. (e) *I*_on_/*I*_off_ and threshold voltage as functions of IZO precursor concentrations. (f) Hole and electron mobilities of vertical heterojunction transistors at various IZO precursor concentrations.

Subsequently, to investigate the influence of the IZO layer on the electrical properties, we fabricated devices with three different IZO precursor concentrations. As shown in Figure 2d, the transfer curves reveal that increasing the IZO precursor concentration substantially suppressed hole accumulation. Both the threshold voltage and the *I*_on_/*I*_off_ ratio exhibited a clear dependence on the IZO precursor concentration (Figure 2e). Furthermore, as shown in Figure 2f, the hole mobility decreased significantly with higher IZO precursor concentration, while the electron mobility showed a slight increase. These results confirm that the origin of the *V*_G_‐independent *I*_D_ phenomenon lies in the properties of the IZO layer.

To understand the mechanisms behind the *V*_G_‐independent *I*_D_ phenomenon, we characterized the surface potential of IZO layers with and without SAM modification using Kelvin probe force microscopy (KPFM) (Figure 3a,b). As shown in Figures 3c and S8, the SAM‐modified IZO layers exhibited a surface potential approximately 0.3 eV higher than that of the unmodified IZO layers, indicating the presence of an interfacial dipole with its positive end oriented toward the IZO layer and its negative end toward the SAM. Based on the KPFM results and previously reported band properties [25, 29, 30], we propose a graphical representation of the predicted energy band alignment for the vertical heterojunction with and without SAM modification (Figure 3c,d). As illustrated in Figure 3e, in the absence of SAM modification, the work function of the IZO layer is lower than that of the DPA layer, forming a typical type‐II heterojunction. Under these conditions, holes injected into the DPA layer subsequently undergo drift and diffuse toward the IZO layer. However, because holes are the minority carriers in IZO, their transport is severely limited. In contrast, when a Me‐4PACz SAM is introduced at the DPA/IZO interface (Figure 3f), the interfacial dipole modifies the local energy landscape. The resulting electric field opposes the drift and diffusion of holes into the IZO layer, leading instead to hole accumulation and rapid saturation within the DPA layer. Moreover, as shown in Figure S9, the vertical heterojunction transistor without SAM modification exhibits clear light emission, whereas the SAM‐modified device shows no detectable emission under identical conditions. This result indicates that, in the absence of the SAM, holes can readily inject from the DPA layer into the IZO layer, where electrons are the majority carriers. These injected holes and electrons then recombine at the DPA/IZO interface, giving rise to light emission. In contrast, when the Me‐4PACz SAM is introduced, the interfacial dipole creates an energetic barrier that effectively confines holes within the DPA layer, preventing their injection into the IZO layer and thereby suppressing electron–hole recombination and the associated light emission. These results indicated that the SAM not only passivates surface defects but also spatially confines hole carriers.

**FIGURE 3:**
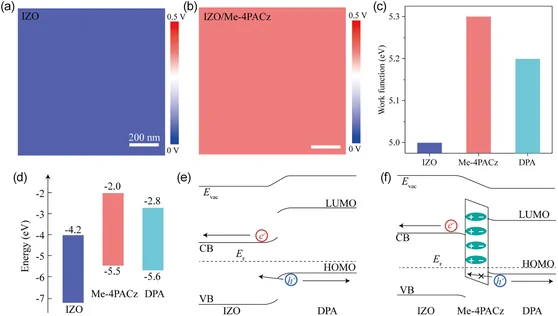
(a) KPFM image of IZO. (b) KPFM image of SAM‐modified IZO. (c) Work function values of IZO, Me‐4PACz, and DPA films. (d) Energy‐level diagrams of IZO, Me‐4PACz, and DPA. (e) Energy band alignments of IZO and DPA vertical heterojunction without and (f) with Me‐4PACz‐modified interface. *E*_vac_ and *E*_F_ are the vacuum level and the Fermi level, respectively. CB and VB are the lowest energy level of the conduction band and the highest energy level of the valence band of the IZO, respectively. LUMO and HOMO are the lowest unoccupied molecular orbital and the highest occupied molecular orbital of DPA, respectively.

To implement a ternary inverter, a vertical heterojunction transistor and a DPA TFT are connected in series, as depicted in the equivalent circuit diagram in Figure 4a. The supply voltage (*V*_DD_) is applied to the drain electrode of the DPA TFT, and the input voltage (*V*_IN_) is applied to the common gate electrode. The source electrode of the vertical heterojunction transistor is grounded, and the output voltage (*V*_OUT_) was monitored at the shared central electrode. The transconductance (*g*_m_) obtained from the typical transfer curves of the DPA TFT and vertical heterojunction transistors with varying IZO precursor concentrations are presented in Figure 4b. As a result of the *V*_G_‐independent *I*_D_, the transfer curves the vertical heterojunction transistor and the DPA TFT overlap only within the subthreshold regime. Given this performance profile, vertical heterojunction transistors fabricated using a 0.2 M IZO precursor concentration were employed to realize the ternary inverter. As shown in Figure 4c, the ternary inverter operation at *V*_DD_ = 20 V is explained by the transfer characteristics of the constituent transistors. For −40 V < *V*_IN_ < −30 V, the drain current of the DPA TFT (*I*_D‐p_) significantly exceeds that of the vertical heterojunction transistor (*I*_D‐n_), establishing a conductive path between the supply and the output electrode. Consequently, *V*_OUT_ ≈ *V*_DD_ (logic 2, Figure 4d). As the *V*_IN_ increased, both *I*_D‐n_ and *I*_D‐p_ decrease monotonically. When *I*_D‐p_ becomes comparable to *I*_D‐n_, an intermediate output voltage is generated (logic 1). Finally, for *V*_IN_ > −15 V, *I*_D‐p_ decreased, but *I*_D‐n_ increased. Thus, *I*_D‐p_ becomes negligible relative to *I*_D‐n_, establishing a conductive path between the output electrode and the ground. This results in *V*_OUT_ ≈ 0 V (logic 0). This sequence of three distinct logic states enables ternary inverter operation. The behavior of the intermediate logic state (logic 1) depends on the magnitude of the supply voltage. As the *V*_DD_ increases, the range of the intermediate output voltage (logic 1) narrows. This occurs because *I*_D‐n_ saturates more rapidly with increasing *V*_DD_. Consequently, the second transition peak (associated with the logic 1 to logic 0 transition) moves closer to the first transition peak (associated with the logic 2 to logic 1 transition). As shown in Figure S10, two distinct peak values are observed in the inverter's gain profile at various *V*_DD_. At *V*_DD_ = 35 V, the second transition peak merges with the first peak, demonstrating that the intermediate logic state is significantly compressed. Notably, when *V*_DD_ deviates from this optimized condition, whether to lower or higher values, the intermediate state disappears, and the device operates as a binary logic gate (Figure S11). This electrical tunability highlights the adaptability of the device to MVL systems. Furthermore, to evaluate the stability of the ternary logic gates, symmetric voltage transfer characteristics with a measured noise margin of 21% of the ideal static noise margin are demonstrated in Figure S12. The ideal margin is given by √2 × *V*_DD_/4 (e.g., 8.84 V for *V*_DD_ = 25 V). This moderate experimental noise margin is likely related to the devices operating in the subthreshold regime and can be further improved by device optimization.

**FIGURE 4:**
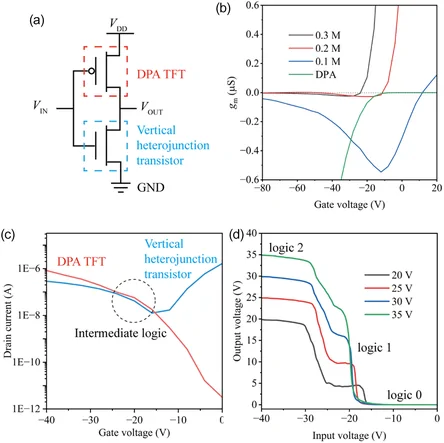
(a) Equivalent circuit of the organic ternary inverter based on a DPA TFT and a vertical heterojunction transistor. (b) Transconductance characteristics of the vertical heterojuciton transistor at various IZO precursor concentrations and DPA TFT. (c) Transfer characteristics of the DPA TFT and vertical heterojunction transistors measured at *V*_DS_ = 20 V. (d) Voltage transfer characteristics of the ternary inverter at various supply voltages.

The ambipolar transistor represents a classical device architecture, whose characteristic V‐shaped transfer curve arises from the competing contributions of electrons and holes—a phenomenon that can conceptually be viewed as a form of NDT. However, in contrast to previously reported MVL designs that rely on pronounced N‐shaped NDT characteristics (often achieved through lateral heterojunctions), our work introduces a simple approach based on a vertical heterojunction with intentionally suppressed hole conduction. This vertical integration yields a simplified structure that inherently avoids the need for complex multithreshold voltage engineering or precise alignment of lateral patterns, thereby streamlining fabrication and enhancing suitability for scalable integration. Moreover, conventional N‐shaped NDT devices generate intermediate states in the above‐threshold regime, relying on substantial current modulation to form a negative transconductance region. Our device, conversely, operates in the subthreshold regime to establish intermediate logic levels. This subthreshold operation substantially reduces the static current and thus improves energy efficiency. This vertical approach presents a more streamlined and promising pathway toward energy efficient and scalable MVL.

## 3 Conclusion

In summary, we demonstrate a novel MVL transistor based on a vertical heterojunction architecture, featuring a solution‐processed IZO n‐type layer and a vacuum‐deposited DPA p‐type layer with a Me‐4PACz SAM‐modified interface. The SAM serves dual critical functions: passivating IZO surface defects and confining hole carriers within the DPA layer. This enables V‐shaped transfer characteristics with suppressed hole conduction and facilitates stable intermediate logic states in an ambipolar transistor. Subsequently, we realize a ternary inverter exhibiting three well‐defined logic states. Notably, the range of the intermediate state is dynamically tunable via supply voltage modulation in the subthreshold regime. This work overcomes some longstanding challenges in MVL implementation, including fabrication complexities of lateral heterostructures (precise material alignment and patterning) and multithreshold instability, through improved vertical integration and tailored interfacial chemistry. This device architecture may show potential in energy‐efficient computing for AI and IoT applications.

## 4 Experimental Section

### 4.1 Materials

Indium nitrate hydrate (In(NO_3_)_3_·*x*H_2_O) and zinc acetate dihydrate (Zn(CH_3_COO)·2H_2_O) were purchased from Sigma–Aldrich. (4‐(3,6‐Dimethyl‐9*H*‐carbazol‐9‐yl)butyl)phosphonic acid (Me‐4PACz) was purchased from Xian Yuri Solar Co., Ltd. 2,6‐Diphenylanthracene (DPA) was purchased from TCI. All chemicals were used directly as received.

### 4.2 Device Fabrication

All devices, including transistors and ternary inverters, were fabricated on highly doped p‐type silicon (Si) substrates coated with a 300‐nm‐thick SiO_2_ layer. The Si wafer pieces underwent successive cleaning steps in deionized water, hot acetone, and isopropanol, followed by drying under a nitrogen stream. An IZO precursor solution (In:Zn molar ratio = 2:1, dissolved in deionized water) was spin‐coated onto the cleaned substrates at 5000 rpm for 60 s. The deposited films were then prebaked on a hotplate at 150 °C for 10 min for solvent removal, followed by annealing at 350 °C for 40 min under ambient conditions. SAMs were formed by spin‐coating a Me‐4PACz solution (0.5 mg/mL in ethanol) onto the IZO thin films at 4000 rpm for 30 s, with subsequent thermal annealing at 100 °C for 10 min. DPA thin film was deposited via thermal evaporation at a rate of 0.1 Å/s to a thickness 50 nm. Finally, 80 nm thick gold source and drain electrodes were patterned using a shadow mask and deposited by thermal evaporation. The devices featured a fixed channel length of 50 μm and channel width of 4 mm. The fabrication procedure for the DPA TFT was nearly identical to that of the vertical heterojunction transistor, except that the DPA TFT did not include the spin‐coated IZO layer. For the ternary logic gates, the vertical heterojunction transistor and the DPA TFT were electrically connected via wire bonding using Au wires.

### 4.3 Characterization

The morphological characteristics of the DPA thin film and the IZO film were determined using an AFM (Vecco Co.) with a tapping mode. The XRD patterns of the samples were collected by an X‐ray diffractometer (Rigaku Smart lab 9 KW) using Cu Kα_1,2_ radiation (wavelength *λ *= 1.541 Å). KPFM images of the perovskite samples deposited on Si wafer were obtained by atomic force microscopy (Cypher ES). FET characteristics were measured using a Keithley 4200A‐SCS semiconductor parameter analyzer with a micromanipulator probe station at room temperature. The optical images were measured using a Dhyana 400D CCD camera.

## Funding

This study was supported by National Natural Science Foundation of China (62274144).

## Conflicts of Interest

The authors declare no conflicts of interest.

## Supporting information

Supplementary Material

## Data Availability

The data supporting the findings of this study are available within the article and its Supporting Information. Additional data are available from the corresponding authors upon reasonable request.
