# Supplementary material for: Ambipolar Transistors Based on Vertical Heterojunctions for Multivalued Logic Gates
Source: Small Sci. 2026 Nov 4;6(11):e70389. doi: 10.1002/smsc.70389 (PMC13733378; doi:10.1002/smsc.70389)
Supplement: Supplementary file 1 [file SMSC-6-e70389-s001.pdf]

Supporting Information

Ambipolar transistors based on vertical heterojunctions for multi-valued logic gates

Ke Zhou, Shengnan Liu, Yucai Yuan, Jiawei Hong, Zhixiang Ren, Yaxiao Lian, Xiaotao Zhang, Wentao Xiong, Chen Zou, Baodan Zhao*, Wenping Hu* and Dawei Di**

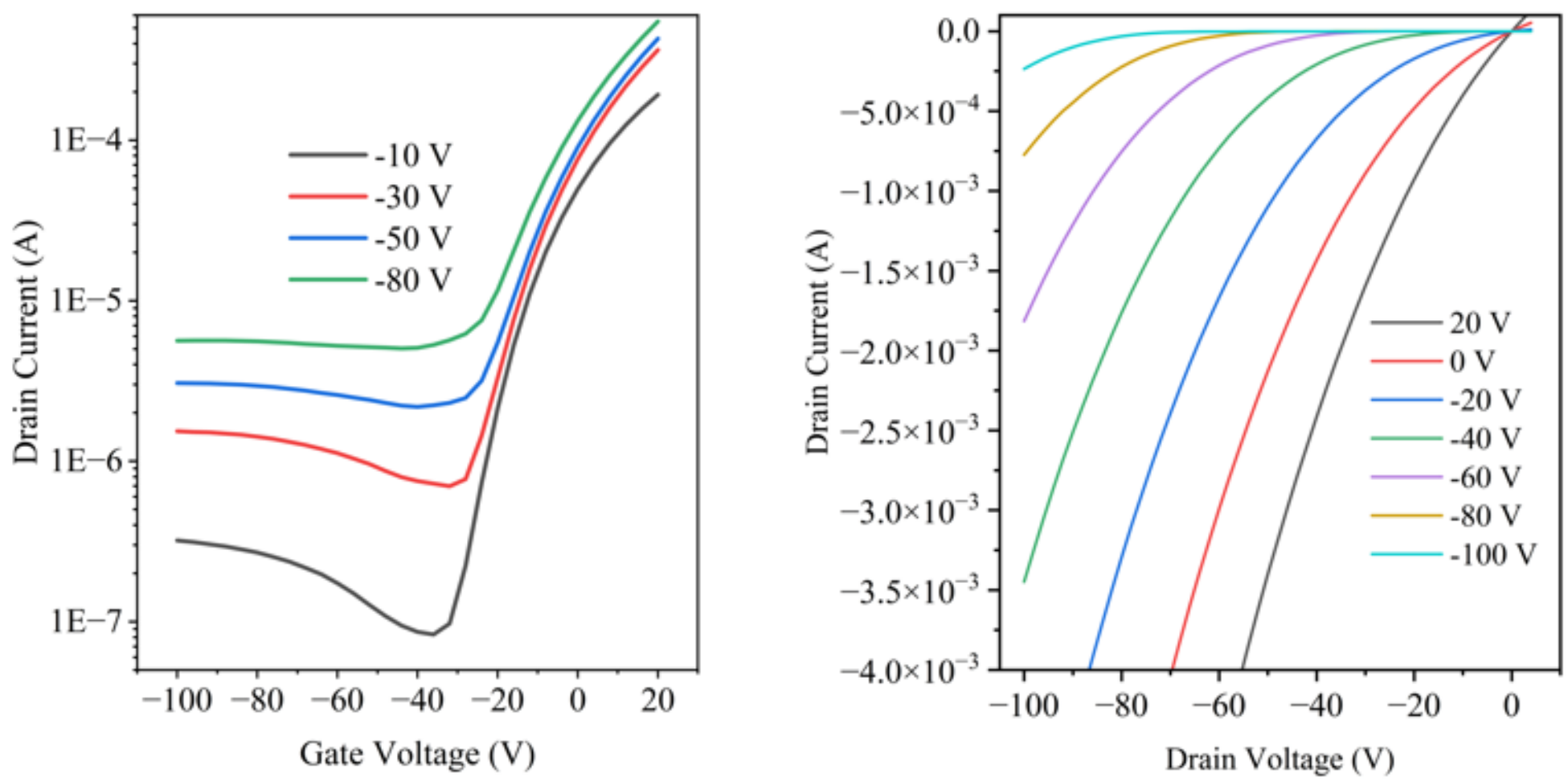

Figure. S1 Typical p-type transfer curves (left) and output characteristics (right) at varying drain-source voltages.

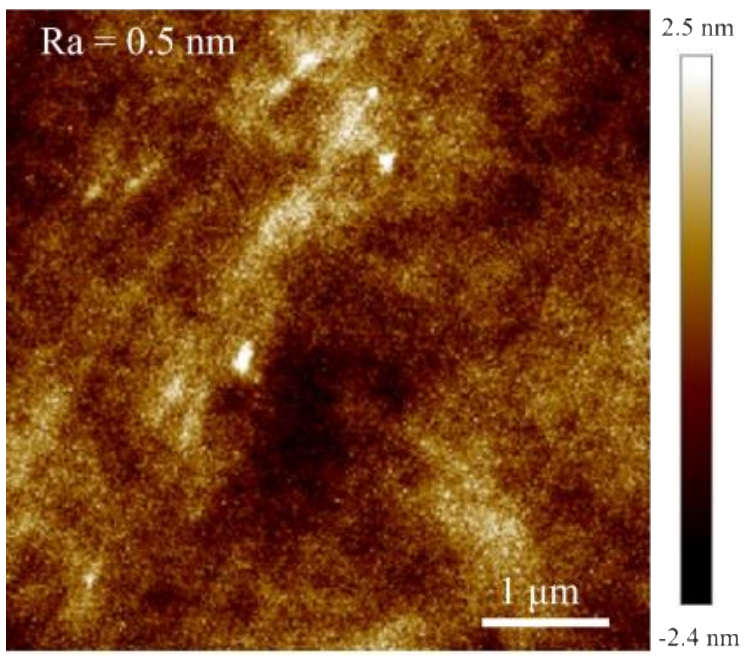

Figure. S2 The AFM image of IZO film.

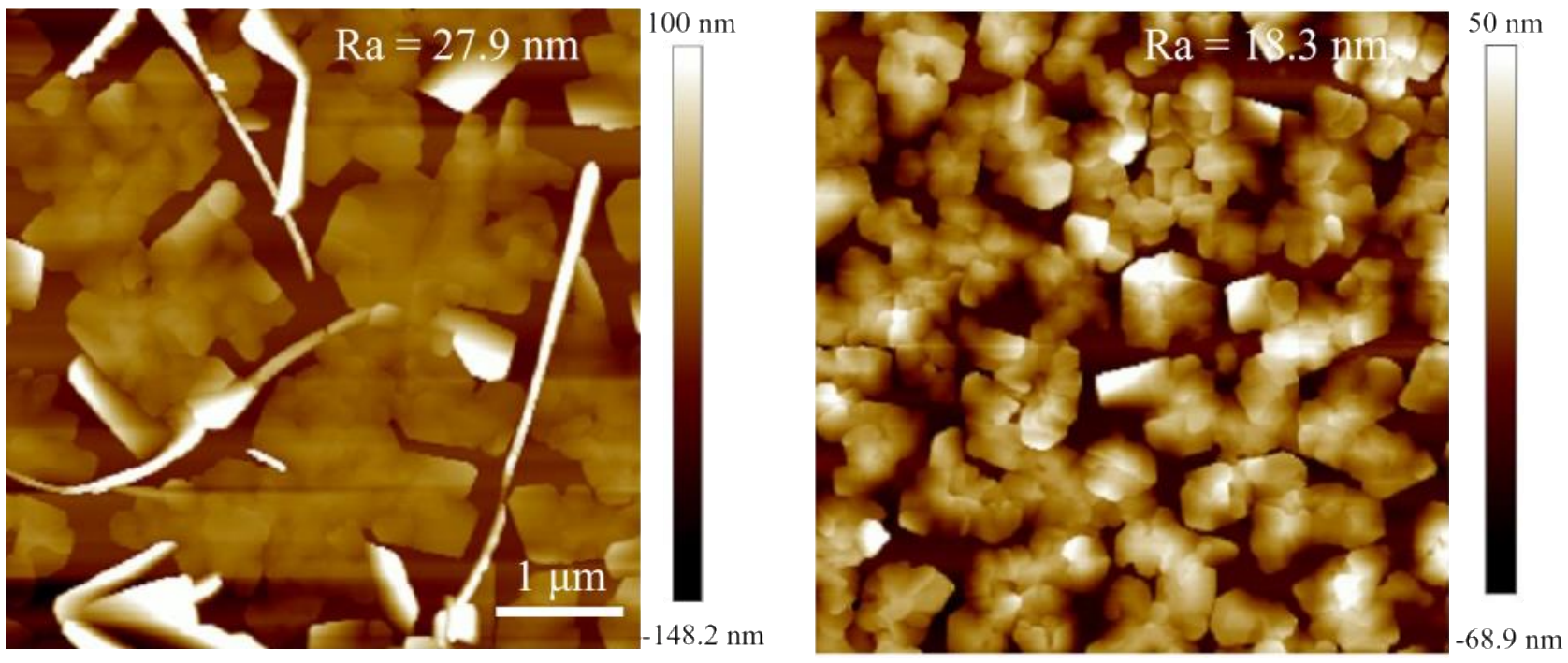

Figure. S3 The morphology of DPA thin films grown on (left) bare IZO versus (right) SAMs-modified IZO surfaces.

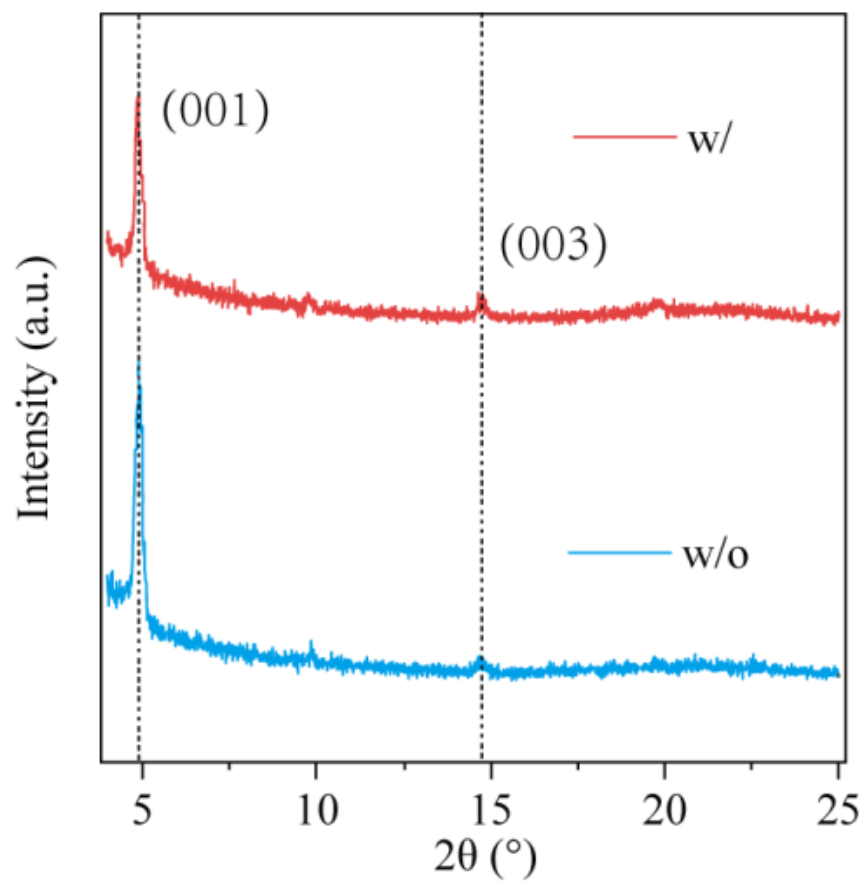

Figure. S4 XRD profiles of DPA thin films grown on IZO and SAMs-modified IZO substrates.

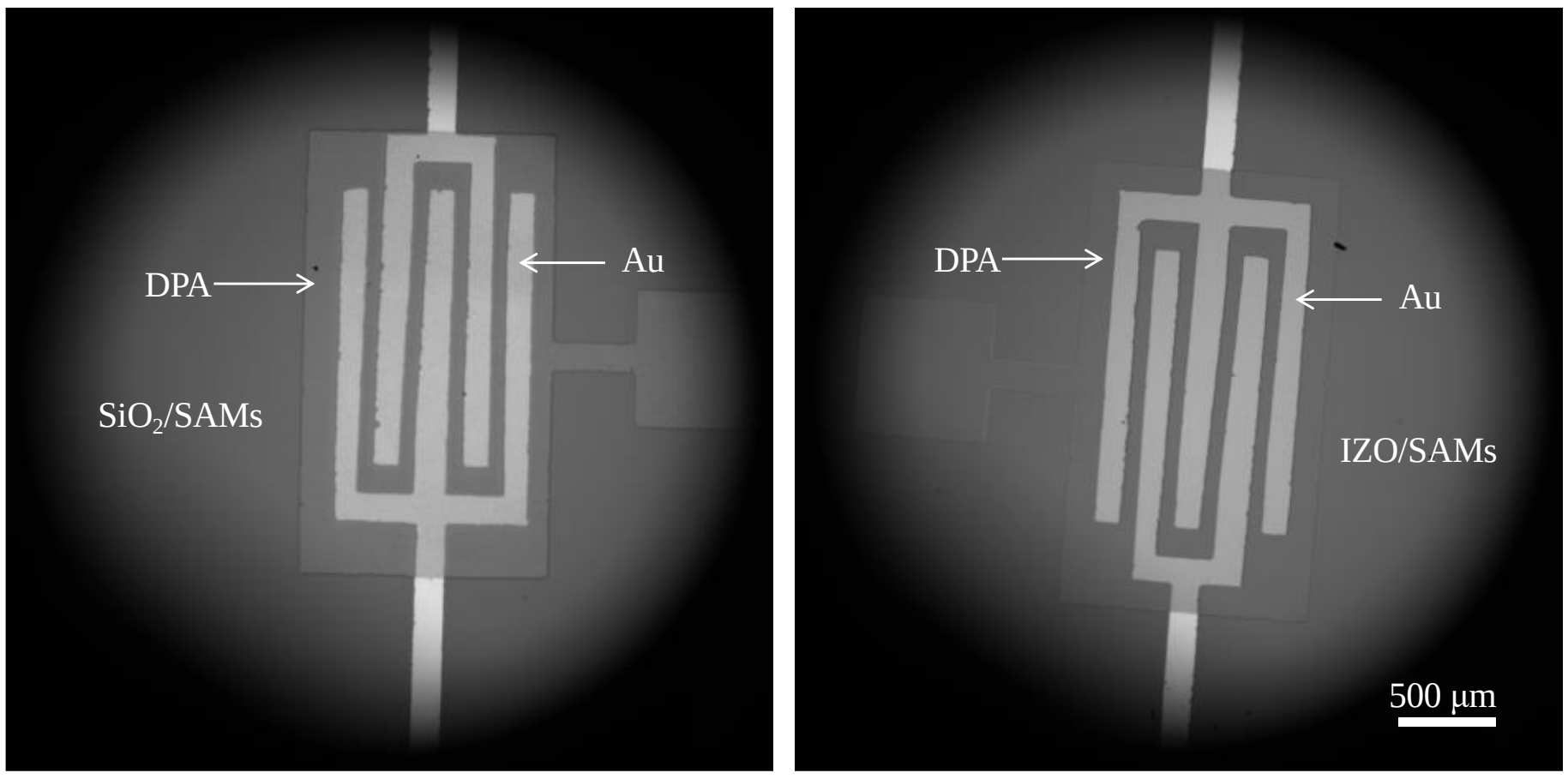

Figure. S5 The optical image of the DPA TFT (left) and the vertical heterojunction transistor (right).

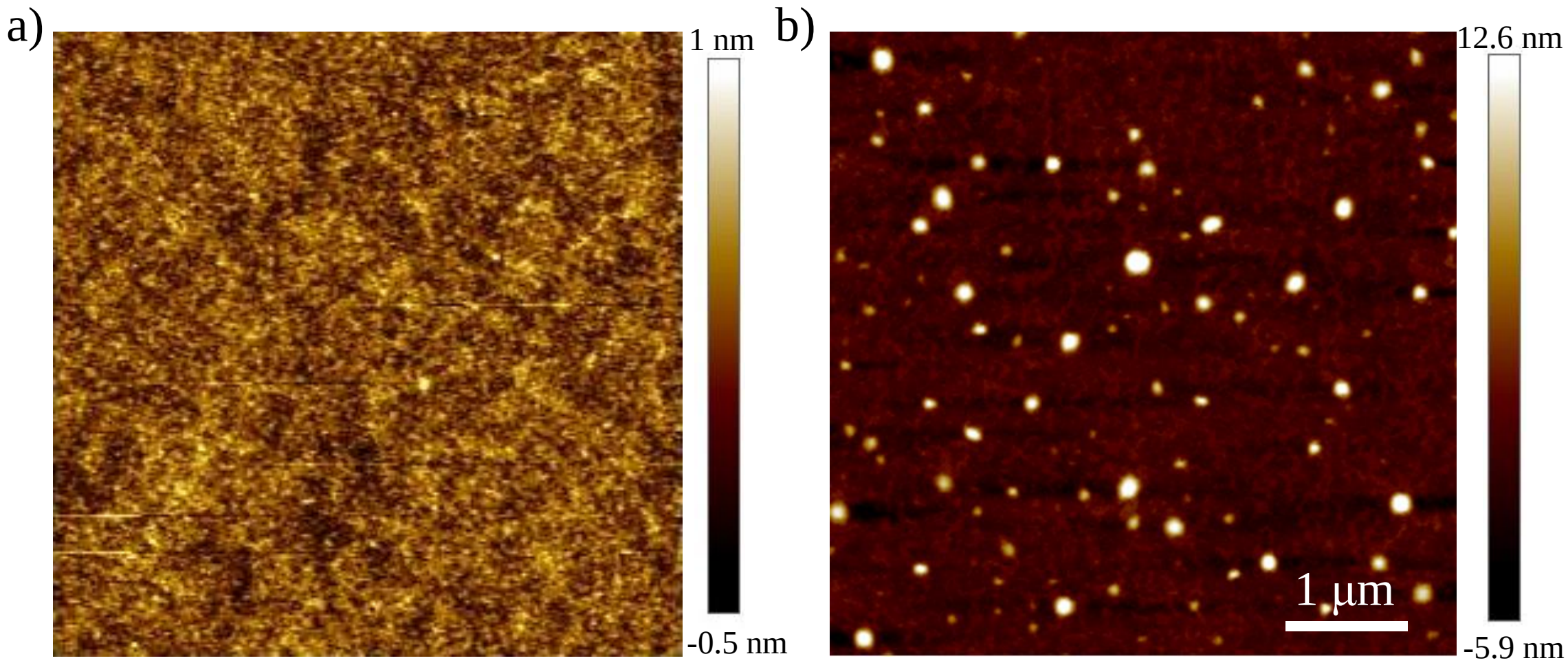

Figure. S6 The morphology of IZO modified with various SAM concentrations. a) 0.1 mg/mL, b) 0.5 mg/mL.

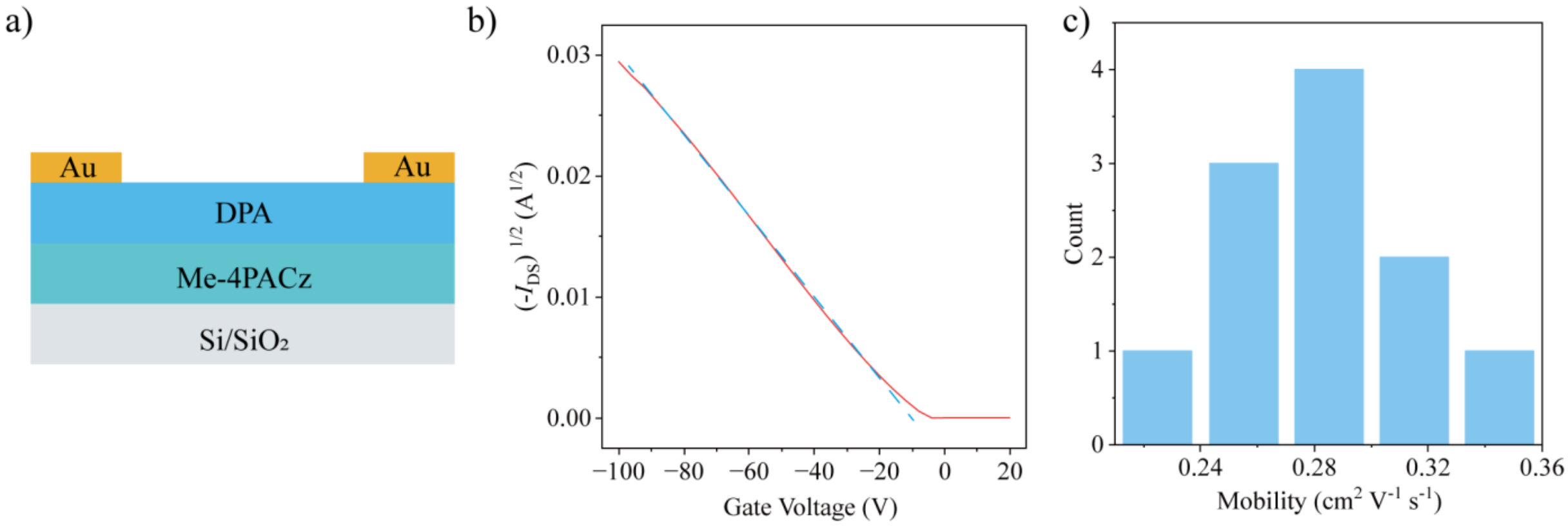

Figure. S7 a) Schematic of the DPA transistor with SAM-modified substrate. b) typical transfer curve of DPA transistor. c) Statistical distribution of mobilities extracted from multiple devices.

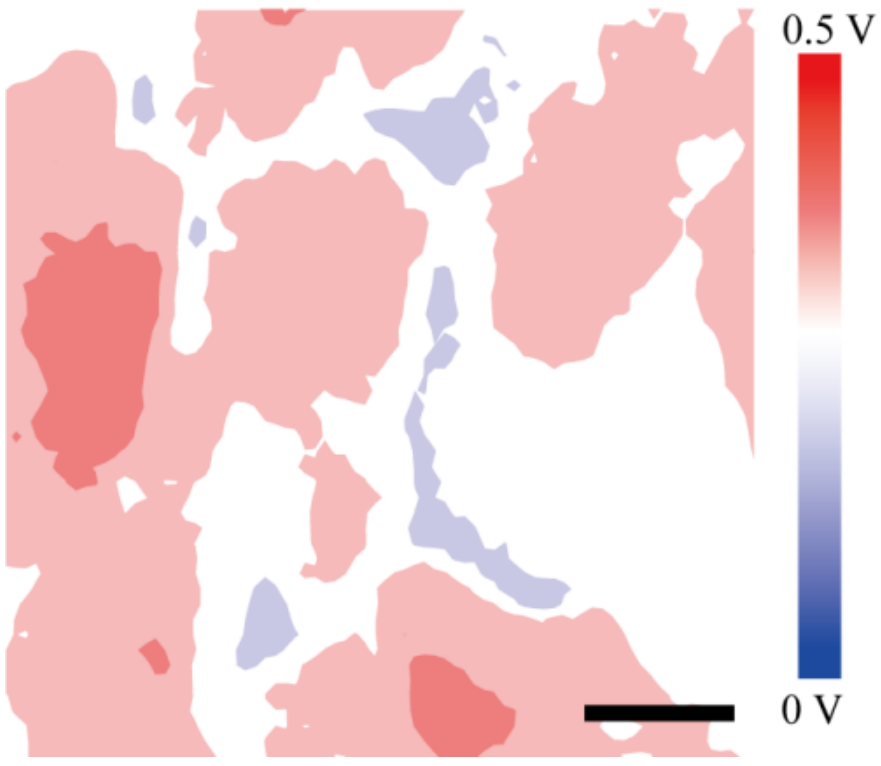

Figure. S8 KPFM image of DPA thin film, scale bar is 200 nm.

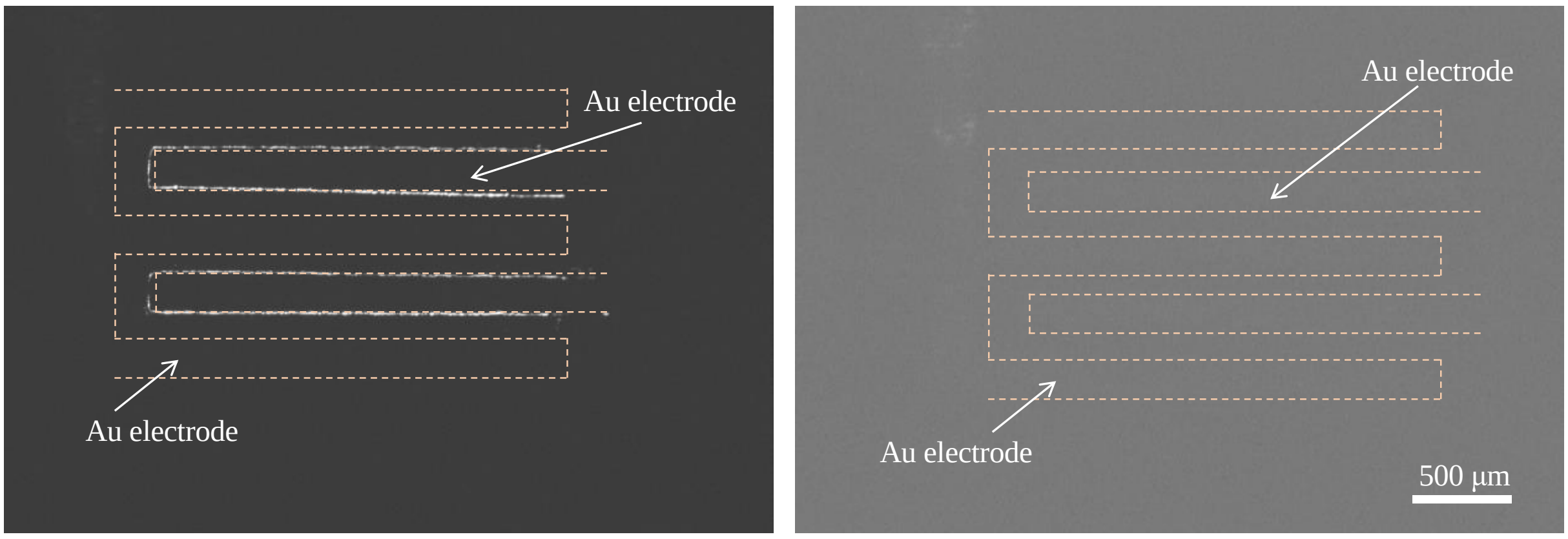

Figure. S9 a) Optical image of light emission from a vertical heterojunction transistor without SAM modification. b) Optical image of a SAM-modified vertical heterojunction transistor under identical bias conditions.

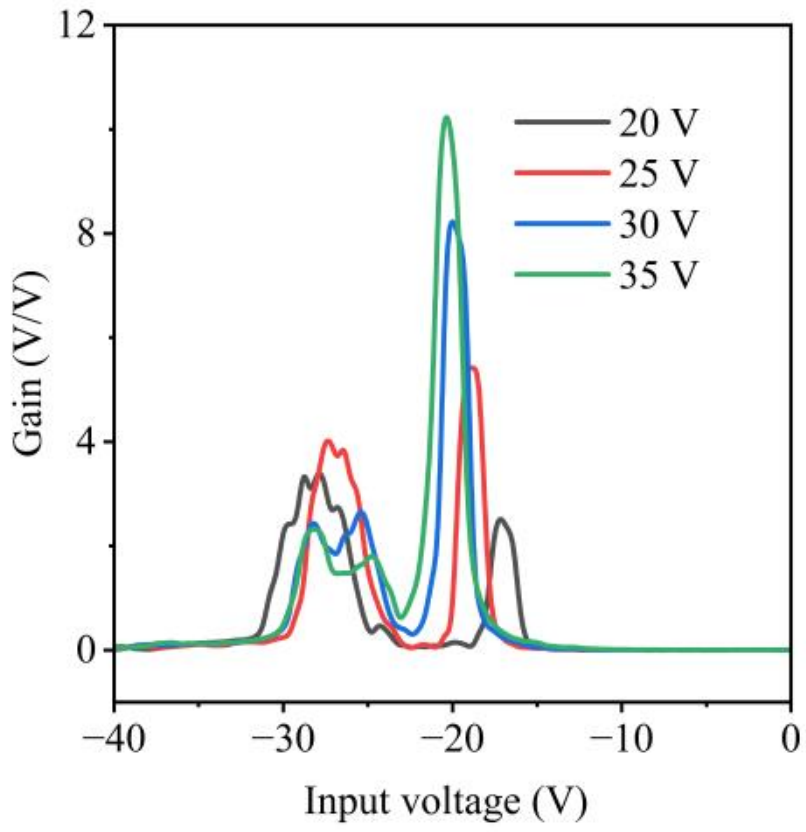

Figure. S10 DC voltage gain of the ternary inverter at various supply voltages.

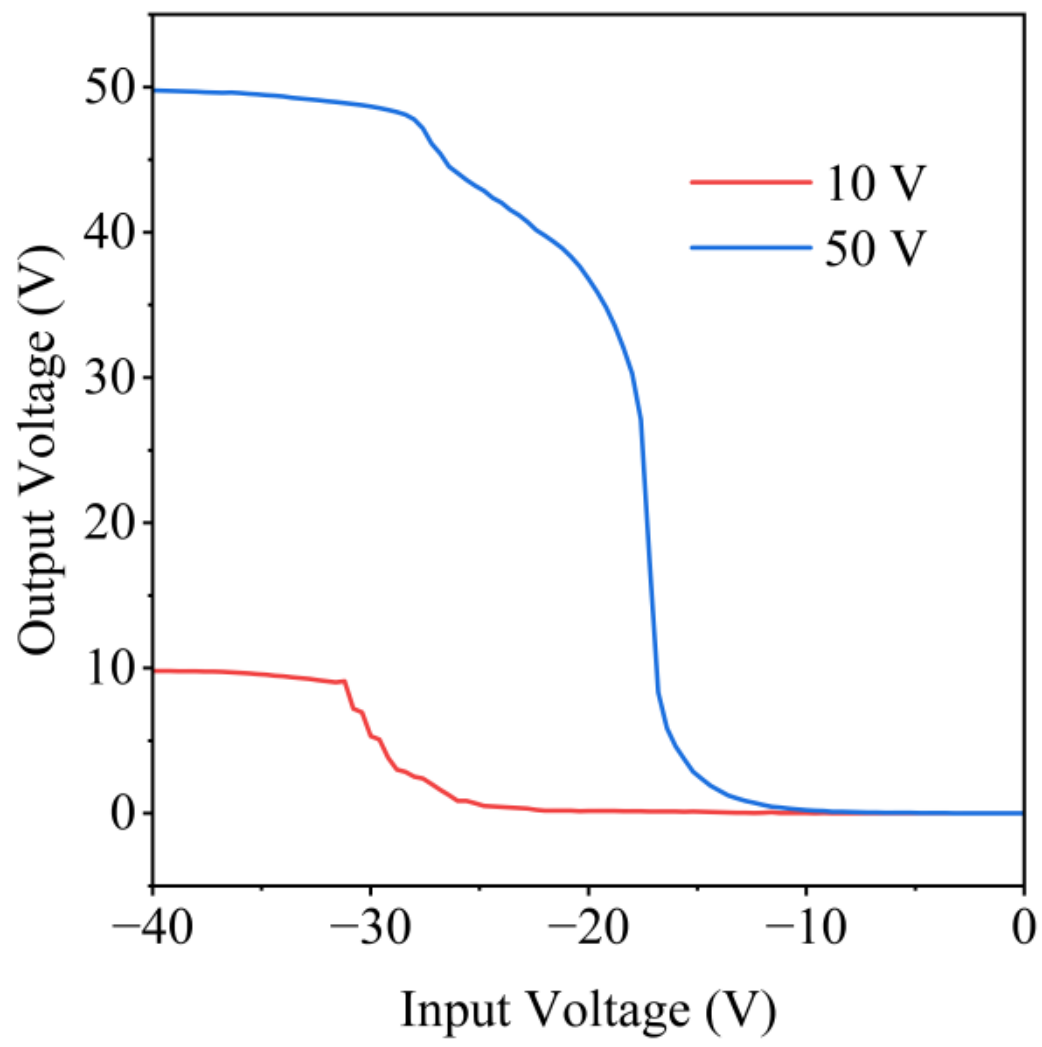

Figure. S11 Voltage transfer characteristics at V_{DD} = 10 V and 50V.

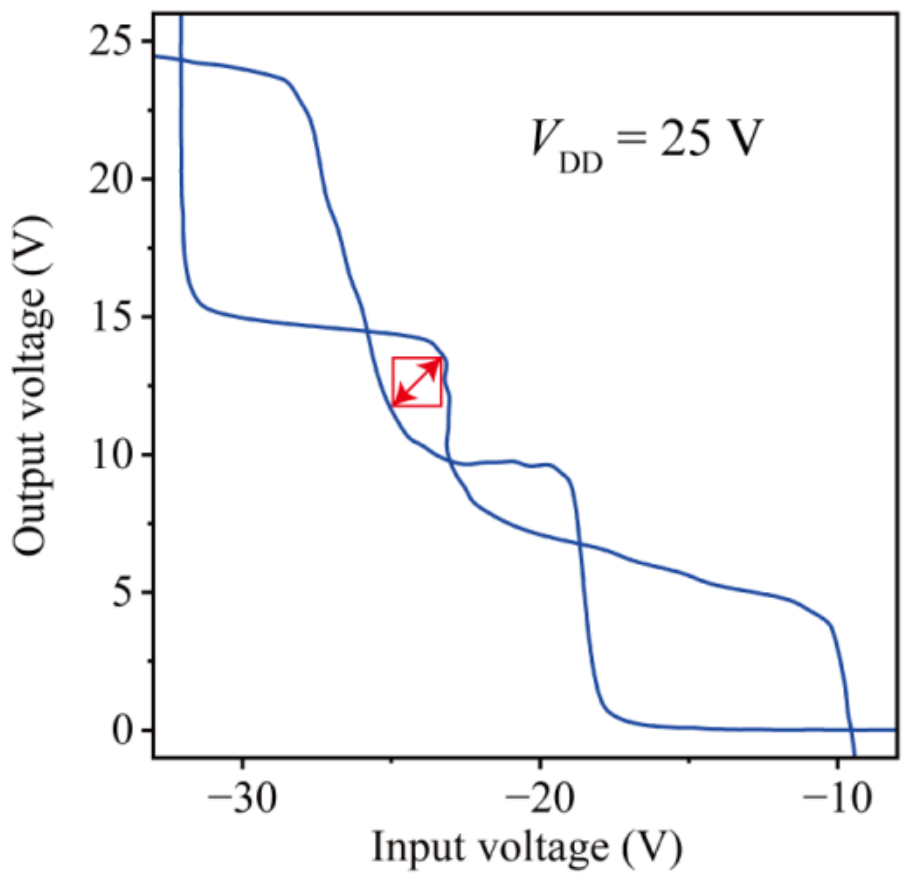

Figure. S12 Butterfly voltage transfer characteristics for static noise margin.
